# Real-World Barriers to and Facilitators of Implementing AI-Based Clinical Decision Support Systems: Scoping Review

**DOI:** 10.2196/89834

**Published:** 2026-08-13

**Authors:** Emma Bogner, Abby Thomas, Bishnu Bajgain, Cody van Rassel, Khara Sauro, Joon Lee

**Affiliations:** 1 Data Intelligence for Health Lab, Cumming School of Medicine, University of Calgary, 3280 Hospital Dr NW, Calgary, AB, T2N 4Z6, Canada, 1 403-220-2968; 2 Department of Cardiac Sciences, Cumming School of Medicine, University of Calgary, Calgary, AB, Canada; 3 Department of Surgery, Cumming School of Medicine, University of Calgary, Calgary, AB, Canada; 4 Department of Community Health Sciences, Cumming School of Medicine, University of Calgary, Calgary, AB, Canada; 5 Department of Pediatrics, Cumming School of Medicine, University of Calgary, Calgary, AB, Canada; 6 Department of Oncology, Cumming School of Medicine, University of Calgary, Calgary, AB, Canada

**Keywords:** machine learning, artificial intelligence, AI, clinical decision support, personalized medicine, implementation science

## Abstract

**Background:**

Widespread and sustained uptake of AI-based clinical decision support systems (CDSSs) in real-world health care settings is uncommon, despite their potential to improve patient care and reduce clinician burnout. Although previous studies have examined determinants of implementing AI-based CDSSs, limited evidence has synthesized barriers and facilitators identified during actual clinical implementation and use.

**Objective:**

The objectives of this scoping review were to (1) map and synthesize barriers to and facilitators of implementing AI-based CDSSs in real-world health care settings and (2) draw on this knowledge to inform future implementation strategies.

**Methods:**

Five electronic databases (MEDLINE, Embase, CINAHL, APA PsycInfo, and the Cochrane Library) were searched from inception to May 2022. Eligible studies included primary research describing real-world implementation processes or reporting determinants (barriers and facilitators) of implemented AI-based CDSSs in any health care setting. Studies focused on non-decision support tasks, non-AI CDSSs, patient-facing tools, or development or effectiveness without implementation were excluded. No study design restrictions were applied. Full texts were reviewed to extract explicit statements describing determinants influencing implementation. These determinants were classified as barriers or facilitators and mapped to the Consolidated Framework for Implementation Research (CFIR) by 2 independent reviewers. A qualitative synthesis was conducted.

**Results:**

After removing 4234 duplicate records, 10,875 articles were screened by title and abstract, which excluded 10,355 articles. After further exclusions based on full-text availability, 494 full-text articles were assessed for eligibility, of which 13 met the inclusion criteria. Nine of these studies reported explicit implementation determinants and were included in the CFIR-based synthesis. Studies were primarily conducted in the United States and involved multicenter implementation of machine learning-based CDSSs in critical care and emergency medicine settings. A total of 28 determinants (16 barriers and 12 facilitators) were identified. Barriers were most frequently mapped to the inner setting, innovation, and individuals domains, whereas facilitators were most frequently mapped to the implementation process and innovation domains. Common barriers included limited algorithm interpretability, data quality and management challenges, misalignment with clinical workflows, and insufficient user capability and motivation. Facilitators included early and ongoing assessment of end-user needs, stakeholder engagement, peer endorsement, and robust supporting evidence.

**Conclusions:**

This review identified key determinants influencing the real-world implementation of AI-based CDSSs, highlighting the importance of system design, organizational context, and implementation strategies. However, the small number of studies reporting explicit implementation determinants underscores a critical gap in the literature, suggesting that many real-world implementations do not adequately evaluate or report factors influencing adoption and sustained use. Addressing this gap will be essential for advancing the translation of AI-based CDSSs into routine clinical practice. These findings provide a foundation for developing targeted implementation strategies and emphasize the need for more rigorous, implementation-focused research in real-world health care settings.

## Introduction

AI refers to the capability of computerized systems to perceive, synthesize, and infer information in a manner mimicking the human brain. This can be achieved through symbolic representations of existing knowledge bases (ie, rule-based AI) or purely data-driven approaches, such as machine learning (ML) [1]. In health care, AI can help clinicians personalize patient care by aiding diagnosis, treatment planning, and risk stratification as well as decreasing their cognitive burden (eg, via task automation) [2]. Recent advances in health IT and digital medicine have also allowed AI to emerge as a driver of efficient, accurate, and confident decision-making [3]. Clinical decision support systems (CDSSs) using AI can leverage the large amounts of health data generated during routine practice to provide patient-level insights and optimize care [4].

The emerging potential of AI to improve patient care is demonstrated by an increasing number of studies describing the development of AI-based CDSSs. However, these tools often do not see successful adoption and integration into clinical practice. Implementing innovations in health care is complex, primarily due to the broad range of interested parties involved (health care providers, patients, caregivers, etc), criticality of the decisions being made, and strict regulations and standards within the space [5,6]. Several challenges unique to AI-based CDSSs add to this complexity, including concerns about legal accountability; limits to autonomy; and data quality, security, and privacy [7-9]. Understanding these determinants represents a foundational step in developing robust strategies that promote successful implementation and sustained use to realize the full clinical benefit of AI-enabled decision support [6].

Although these issues have been explored extensively in the literature, many studies have focused on implementation planning, feasibility, or anticipated adoption challenges rather than determinants identified during real-world implementation. Hence, this scoping review aimed to (1) map and synthesize barriers to and facilitators of implementing AI-based CDSSs in real-world health care scenarios and (2) draw on this knowledge to inform future implementation strategies.

## Methods

### Design

This scoping review was conducted according to the Joanna Briggs Institute methodology and reported following the PRISMA-ScR (Preferred Reporting Items for Systematic Reviews and Meta-Analyses extension for Scoping Reviews) checklist. Ethics approval was not required as all data used were published prior to study initiation.

### Data Sources, Search Strategy, and Article Selection

A detailed protocol for this review has been published previously [3]. In brief, potential evidence sources were identified by searching 5 electronic databases (MEDLINE, Embase, CINAHL, APA PsycInfo, and the Cochrane Library) for articles describing clinical decision support, implementation and/or determinants, and AI or ML published from database inception until May 10, 2022. These databases were selected to capture clinical and health service literature relevant to the real-world implementation of AI-based CDSSs. Given the focus on implementation within clinical practice environments, databases indexing biomedical and health service research were prioritized. Technical and engineering databases were not included as they predominantly index studies focused on algorithm development, model performance, or technical validation rather than real-world clinical implementation.

Key search concepts and corresponding keywords are listed in Table 1. The full electronic search strategy for MEDLINE (Ovid) is provided in Table S1 in Multimedia Appendix 1 [10-18]. Search strategies for the other databases were adapted from this core strategy using database-specific operations and syntax. The titles and abstracts of the articles returned from the search were screened for potential relevance by 3 independent reviewers, requiring agreement from at least 2 reviewers to be included for full-text screening. The full-text screening and selection of final articles was carried out by 2 independent reviewers according to the eligibility criteria described in the following section. Disagreements were handled by a third reviewer who participated in the initial abstract screening. Reasons for exclusion at the full-text stage were recorded and are summarized in the PRISMA (Preferred Reporting Items for Systematic Reviews and Meta-Analyses) flow diagram.

**Table 1.** Search concepts and keywords for initial database search strategy.

Concepts^a^	Relevant keywords
Clinical decision support	exp Decision Support Systems, Clinical/ OR Decision-support* OR “Clinical support system” OR “clinical decision support system” OR “Clinical decision support” OR “evidence-based” OR “evidence-based support” OR “support system” OR exp Clinical protocols OR clinical guideline* OR clinical guidance* OR medical guideline* OR medical guidance*
Implementation barriers and facilitators	implement* OR “implementation strategy” OR strateg* OR barrier* OR enabler* OR facilitator* OR determinant* OR satisfaction* OR perception* OR experience*
AI and machine learning	“Artificial intelligence” OR “AI” OR “Machine Learning” OR “ML” OR “Deep Learning” OR “Augmented Intelligence” OR “Reinforcement Learning” OR “Neural Network” OR Unsupervised Machine Learning” OR “Supervised Machine Learning” OR “Random Forest” OR “Support Vector Machine” OR “Decision Tree” OR “Classification” OR “Rule-based” OR “Symbolic Artificial Intelligence” OR “Symbolic AI”

a Concepts were combined using the Boolean and proximity operator “AND,” and the search terms within each concept were combined using “OR.”

### Eligibility Criteria

Primary research describing the implementation process or reporting determinants (barriers and facilitators) of implemented AI-based CDSSs was considered eligible for inclusion. AI was defined as any computerized system with capabilities for perceiving, synthesizing, and inferring information in a humanlike manner, encompassing both traditional rule-based and modern data-driven techniques [1]. Clinical decision support was defined as the generation of actionable diagnostic or prognostic insights intended to aid clinicians in making personalized decisions about patient care, which resulted in the exclusion of studies focusing on nondecision task automation, for example, image segmentation and recognition tasks in radiology. Studies were also excluded if they described non-AI-based CDSSs or solely patient-facing aids or if they reported on the development and/or effectiveness of AI-based CDSSs but did not describe their implementation. Eligibility was not limited by study design or language. A summary of the inclusion and exclusion criteria is provided in Table 2.

**Table 2.** Eligibility criteria for identifying studies on the implementation of AI-based clinical decision support systems (CDSSs).

Domains	Inclusion criteria	Exclusion criteria
Population and setting	Clinical health care settings involving patient care	Nonclinical or purely simulated settings
Intervention	AI-based CDSSs	Non-AI CDSSs or tools not intended for clinical decision support
CDSS function	Provides treatment planning or prognostic decision support	Limited to nondecision tasks (eg, segmentation and data processing)
Implementation focus	Reports implementation process or determinants (barriers and facilitators)	Model development, validation, or performance evaluation only
End user	Intended for clinician use	Patient-facing decision aids
Study design	All study designs	None (no design restrictions)

### Quality Assessment

Consistent with scoping review methodology and the objective of mapping the breadth of available evidence, a formal assessment of risk of bias or methodological quality of the included studies was not conducted.

### Data Extraction and Analysis

Bibliometric information, study characteristics, reported determinants, and implementation details were abstracted from the articles selected for inclusion using a standardized data extraction form developed and piloted by 2 independent reviewers. Prior to full data extraction, reviewers completed a calibration exercise using a subset of included studies to ensure consistency in data abstraction. A split-half approach was used whereby each reviewer extracted data from a subset of studies and cross-checked the extracted data to identify and resolve discrepancies.

All abstracted determinants were mapped to the updated Consolidated Framework for Implementation Research (CFIR) to inform our analysis of implementation experiences [19]. The CFIR was selected as it provides a comprehensive and widely used framework for systematically categorizing multilevel determinants influencing implementation in health care settings [19,20]. The CFIR contains 5 main domains: innovation, inner setting, outer setting, individuals, and implementation process. The innovation domain refers to characteristics of the system being implemented. The inner setting and outer setting domains refer to the setting in which the innovation is being implemented (eg, clinical unit or department) and the setting in which this inner setting exists (eg, hospital or health system), respectively. The individuals domain refers to characteristics of the individuals involved in implementation (eg, leaders, innovation deliverers, and innovation recipients), and the implementation process domain describes the activities and strategies being used to implement the innovation.

Determinants were identified by reviewing the full texts of the included studies to extract explicit statements describing factors that influenced the implementation of AI-based CDSSs. Extracted data consisted of verbatim quotations or clearly stated author interpretations reflecting barriers to or facilitators of implementation.

Two reviewers independently identified and extracted eligible determinant statements and classified them as barriers or facilitators based on whether they were described as hindering (barriers) or supporting (facilitators) implementation processes or outcomes. These determinants were then mapped to CFIR domains and constructs. Mapping decisions were based on the underlying meaning and implementation context of each extracted statement, with determinants assigned to the CFIR construct that most closely reflected the primary implementation-related factor described. General descriptions of system features, usability outcomes, or design characteristics were not considered determinants unless they were explicitly linked to implementation processes or outcomes. Discrepancies were resolved through discussion and consensus. Only studies reporting explicit implementation determinants were included in the CFIR-based synthesis.

## Results

### Study Characteristics

**Figure 1.**
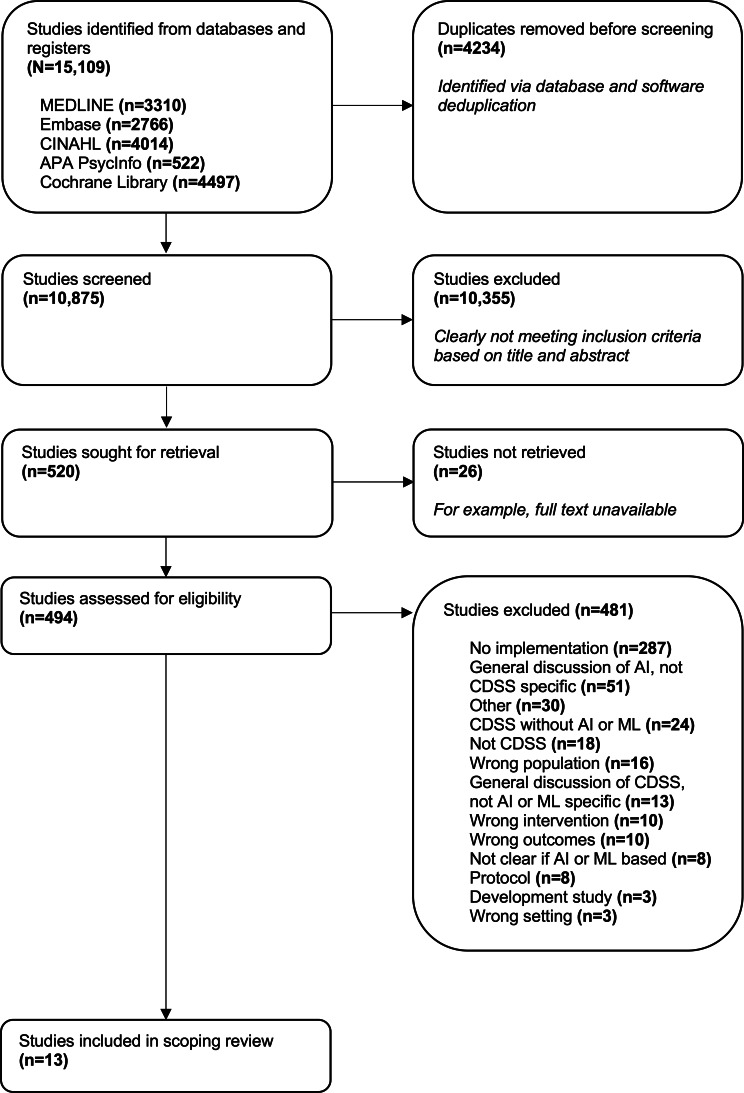
PRISMA (Preferred Reporting Items for Systematic Reviews and Meta-Analyses) flow diagram illustrating the identification, screening, eligibility, and inclusion of studies in this scoping review. The diagram includes database-specific retrieval counts for each source (MEDLINE, Embase, CINAHL, APA PsycInfo, and Cochrane Library) and brief justifications for exclusions at each stage of the screening process, with detailed reasons for exclusion at the full-text review stage. CDSS: clinical decision support system; ML: machine learning.

A total of 15,109 evidence sources were identified in our initial search, of which, after removal of 4234 (28.0%) duplicate records, 10,875 (72.0%) studies were screened based on title and abstract. Following title and abstract screening, of the 10,875 screened studies, 10,355 (95.2%) were excluded. After removing based on full text availability, 494 (4.5%) articles were assessed for eligibility. Of these 494 articles, 13 (2.6%) met the inclusion criteria and were included in the final review (Figure 1). Study-level characteristics for each included source of evidence are summarized in Table 3.

**Table 3.** Study-level characteristics of included sources of evidence and inclusion in Consolidated Framework for Implementation Research (CFIR)–based determinant mapping.

Study and year	Country	Study design	Clinical domain	Implementation scale	Decision support algorithm	Funding source	CFIR mapping^a^
Bauer et al [21], 2002	United States	Mixed methods	General medicine	Single center	Non-ML^b^ based	Institutional (Mayo Clinic)	No
Goldstein et al [10], 2004	United States	Experience report	Cardiology	Multicenter	Non-ML based	Government (VA^c^ HSR&D^d^ and NIH^e^)	Yes
Henry et al [11], 2022	United States	Qualitative	Critical care	Single center	ML based	Foundation and government (Gordon and Betty Moore Foundation, NSF^f^, and Sloan Foundation)	Yes
Hinson et al [22], 2022	United States	Quantitative	Emergency medicine	Multicenter	ML based	Government and institutional (AHRQ^g^, CDC^h^, and JHHS^i^)	No
Jauk et al [12], 2021	Austria	Mixed methods	Critical care	Single center	ML based	Mixed (government, institutional, and industry: CBmed or COMET^j^, KAGes, and SAP SE)	Yes
Ji et al [13], 2021	China	Qualitative	Varied	Multicenter	ML based	Government (National Institute of Hospital Administration, China)	Yes
Joshi et al [14], 2022	United States	Qualitative	Critical care	Multicenter	ML based	Government (NIH)	Yes
Miller et al [15], 2019	United States	Mixed methods	Emergency medicine	Multicenter	Non-ML based	Government (NICHD^k^)	Yes
Romero-Brufau et al [16], 2020	United States	Quantitative	Hospital admissions	Single center	ML based	Institutional (Mayo Clinic)	Yes
Romero-Brufau et al [17], 2020	United States	Qualitative	Primary care	Multicenter	Non-ML based	Government (NIH)	Yes
Gonçalves et al [18], 2020	Brazil	Experience report	Critical care	Single center	Non-ML based	Government (CAPES^l^)	Yes
Singer et al [23], 2022	United States	Qualitative	Hospital admissions	Multicenter	ML based	Institutional (MIT^m^ Sloan School of Management)	No
Tsai et al [24], 2022	Taiwan	Experience report	Emergency medicine	Multicenter	ML based	Institutional (Chi Mei Medical Center)	No

a Indicates whether the study was included in the CFIR-based determinant mapping.

b ML: machine learning.

c VA: US Department of Veterans Affairs.

d HSR&D: Health Services Research and Development.

e NIH: National Institutes of Health.

f NSF: National Science Foundation.

g AHRQ: Agency for Healthcare Research and Quality.

h CDC: Centers for Disease Control and Prevention.

i JHHS: Johns Hopkins Health System

j COMET: Competence Centers for Excellent Technologies

k NICHD: National Institute of Child Health and Human Development.

l CAPES: Coordenação de Aperfeiçoamento de Pessoal de Nível Superior

m MIT: Massachusetts Institute of Technology.

Most excluded studies did not report on system implementation at all or did not describe it in sufficient detail (Figure 1). Nearly all included articles (11/13, 85%) were published between 2019 and 2022 by scholars from the United States. Over half (7/13, 54%) of the selected studies discussed AI-based CDSSs developed for use in critical care (ie, sepsis or delirium) and emergency medicine settings, and most systems (8/13, 62%) were ML based and implemented at a multicenter scale (Table 3).

Of the 13 included studies, 9 (69.2%) explicitly reported implementation determinants (barriers and/or facilitators) and were therefore included in the CFIR mapping. The remaining 4 (30.8%) studies did not meet the criteria for explicit determinant extraction as they focused primarily on system design, usability, or general implementation considerations without clearly identifying barriers or facilitators tied to the implementation process. Extracted quotations, CFIR mappings, and barrier and facilitator classifications are provided in Table S2 in Multimedia Appendix 1 [10-18].

### Principal Determinants of Implementation

#### Overview

This synthesis is based on the 9 included studies that explicitly reported determinants (barriers and facilitators) of implementing AI-based CDSSs. A total of 26 quotes were extracted, from which 28 distinct determinants were identified and mapped to the CFIR. Of these 28 determinants, 16 (57.1%) were classified as barriers, and 12 (42.9%) were classified as facilitators. A detailed breakdown of the CFIR domain and construct mappings is provided in Table 4. Full study excerpts with corresponding classifications and mappings are presented in Table S2 in Multimedia Appendix 1 [10-18].

**Table 4.** Breakdown of Consolidated Framework for Implementation Research (CFIR) domain and construct mappings, including studies reporting the associated determinants.

CFIR domains and constructs	Studies
Innovation	
Innovation evidence base	[10,11,14,18]
Innovation complexity	[11,13,14]
Innovation relative advantage	[16,17]
Innovation adaptability	[11]
Inner setting	
Compatibility	[13,14,17]
Structural characteristics	[12,13,18]
Available resources	[18]
Access to knowledge and information	[10]
Culture	[14]
Implementation process	
Assessing needs	[11,15]
Engaging	[10,18]
Teaming	[10]
Planning	[10]
Individuals	
Innovation deliverers	[11,18]
Capability	[10,18]
Motivation	[10,11]
Opinion leaders	[10]
Implementation leads	[10]
Outer setting	
External pressure	[10,11]

Barriers were most frequently mapped to the inner setting domain (6/16, 38%), followed by the innovation (5/16, 31%), individuals (4/16, 25%), and outer setting (1/16, 6%) domains, with no barriers mapped to the implementation process domain. In contrast, facilitators were most frequently mapped to the implementation process domain (6/12, 50%), followed by the innovation (3/12, 25%), inner setting (2/12, 17%), and outer setting (1/12, 8%) domains, with no facilitators mapped to the individuals domain.

Overall, determinants were most commonly mapped to the innovation (8/28, 29%) and inner setting (8/28, 29%) domains, followed by the implementation process (6/28, 21%), individuals (4/28, 14%), and outer setting (2/28, 7%) domains. Within the innovation domain, the evidence base and complexity constructs were most frequently represented, whereas compatibility and structural characteristics accounted for most of the inner setting mappings. Determinants mapped to the implementation process domain were exclusively identified as facilitators, whereas those mapped to the individuals domain were exclusively identified as barriers.

#### Innovation

A large proportion of determinants identified in this study were mapped to the innovation domain, which focuses on the characteristics of the system being implemented [19]. Key determinants within this domain were related to system complexity, the strength of the evidence base, transparency of AI models, concerns related to data quality and consistency, and perceived relative advantage over existing clinical practices. These determinants were reported across multiple studies and reflect common considerations related to the design and functionality of AI-based CDSSs.

#### Inner Setting

Determinants mapped to the inner setting domain, which refers to the setting in which the innovation is implemented (eg, hospital, clinic, or health care organization), were also identified as important when implementing AI-based CDSSs [19]. Key determinants within this domain included availability of IT infrastructure and hardware and data management requirements. Several studies highlighted the importance of integrating new systems into existing clinical practice without disrupting workflows [10,13,14,17].

#### Individuals

A small number of determinants identified in this study were mapped to the individuals domain, which explores the roles and characteristics of those directly involved with the innovation and its implementation [19]. Determinants within this domain included user skills, motivation, and the roles of team leads and opinion leaders, as well as considerations related to assessing user needs and preferences.

#### Implementation Process

Determinants mapped to the implementation process domain, which encompasses the strategies and activities used to plan, execute, and sustain implementation [19], included activities such as planning, engaging stakeholders, and establishing multidisciplinary teams to support implementation.

#### Outer Setting

Determinants mapped to the outer setting domain, which refers to external influences on implementation, such as policies, regulations, and broader health care system factors [19], included the use of periodic feedback on guideline concordance to support sustained clinician engagement. Additional determinants reflected concerns about potential regulatory use of AI-based CDSSs to standardize care in ways that may conflict with clinician judgment, as well as the need for external validation (eg, clinical trial evidence) to support trust in system recommendations. No determinants were mapped to the policies and laws construct of the outer setting domain.

## Discussion

### Principal Results

Despite a recent increase in publications describing the development of AI-based CDSSs, successful real-world implementations in health care remain limited. Given their potential to improve the quality of patient care and reduce clinician burden, a better understanding of how to effectively implement these tools is needed [8].

This scoping review identified several key determinants from studies describing real-world implementations, including the robustness of the evidence base supporting efficacy claims, the availability of sufficient IT infrastructure, how well the system can be integrated with current clinical workflows and processes, and the overall complexity of the system. Assessing clinician needs, outlining and reviewing implementation plans, forming multidisciplinary implementation teams, encouraging clinician adoption and participation in implementation, and providing performance-based feedback were also found to be primary facilitators of implementation. Conversely, barriers may be faced when clinician end users and/or implementation leads lack the capabilities or motivation to fully commit to the project. Other determinants included the generalizability of the AI models to different patient populations, the relative advantage offered by the system compared to current practice, access to training sessions and educational materials for users, data availability, and shared values about clinician and patient needs.

Notably, only a small number of studies reported explicit implementation determinants, highlighting a critical gap in the literature. This suggests that many real-world implementations of AI-based CDSSs may not adequately evaluate or report factors influencing adoption and sustained use. Addressing this gap will be essential for advancing the translation of these systems into routine clinical practice.

### Principal Determinants of Implementation

#### Innovation

Our findings indicate that a large proportion of implementation determinants were related to the innovation domain, highlighting the importance of system-level characteristics in shaping the adoption of AI-based CDSSs. In particular, the complexity and evidence base underpinning these systems appear to play a central role in influencing initial acceptance by clinician end users.

For many clinicians, the importance of practicing evidence-based medicine contributes to a (potentially well-founded) mistrust of black-box AI models making predictions for which credibility cannot be easily confirmed. They may also face additional difficulties in shared decision-making environments where sufficient levels of patient understanding are required [13,25]. This “black-box problem” is increasingly evident for modern ML algorithms, for which a significant trade-off is often made between performance and explainability [25,26].

Clinicians may also have doubts about the accuracy, generalizability, and clinical credibility of AI-based CDSSs [8,11,27]. These reservations are often fueled by concerns surrounding data quality, consistency, and breadth resulting from firsthand experiences with electronic medical record systems and scattered data management strategies [13,28,29].

Even when trust is not a limiting factor, many clinicians will still resist adopting AI-based CDSSs if they do not see them as providing any considerable advantage over their current ways of practicing [10,17].

#### Inner Setting

Determinants related to the inner setting domain were also identified as important in this review, highlighting the role of the organizational context in the implementation of AI-based CDSSs. In particular, factors related to workflow integration, infrastructure, and data management appear to influence the successful adoption of these systems.

Several studies included in this review underlined the importance of properly integrating new systems into existing clinical practice without disrupting workflows [10,13,14,17]. This requirement is echoed again in the literature, which emphasizes that systems requiring additional time and attention for use are more likely to face challenges with initial uptake by end users [27,30].

Implementations of AI-based CDSSs may also suffer in settings without the required IT infrastructure and/or hardware to support system functions [12,13,18]. ML-based systems in particular require large amounts of data for model training, and generating a single inference often requires inputting hundreds of patient variables. This necessitates strict requirements for the collection, standardization, and storage of data, as well as automatic data entry capabilities to avoid workflow disruption and clinician fatigue [31-33].

#### Individuals, Implementation Process, and Outer Setting

Determinants related to the individuals, implementation process, and outer setting domains were less frequently identified in this review but still highlight important considerations for the successful implementation of AI-based CDSSs. In particular, factors related to user capability, motivation, engagement, and broader organizational and system-level influences appear to affect both initial uptake and sustained use of these systems.

AI-based CDSSs are overall more complex to install, maintain, and use than their non-AI counterparts. As such, implementation projects may benefit from selecting team leads who have experience in informatics or IT [10].

Considerable challenges may also be faced if clinician end users feel that they lack the skills to properly understand and incorporate AI recommendations into their guidelines for practice [9,18]. Beyond initial uptake, sustained adoption may also fail if the clinician end users and/or opinion leaders in their circle lack motivation to use the newly implemented system [10,11]. Assessing the priorities, preferences, and needs of the clinician end users prior to implementation can help pre-emptively address trust and workflow integration–related hurdles [10,11,15]. Actively engaging clinician end users to encourage and support adoption can also facilitate successful implementation, and regularly sharing performance-related feedback with individual users, for example, concordance with existing clinical guidelines, can help sustain long-term interest [10,18].

In addition, discussing plans with local hospital administrators and IT staff and establishing multidisciplinary teams to coordinate the process can help identify and remove organizational barriers to successful implementation [10].

Economic and infrastructure-related considerations may also influence the implementation of AI-based CDSSs, particularly across different health care systems and resource settings. Previous literature has identified barriers such as insufficient computing resources, limited IT infrastructure, and inconsistent internet connectivity, challenges that may be especially pronounced in low- and middle-income countries [33,34]. Although these determinants were not explicitly identified in the included studies, their absence may reflect underreporting within the current implementation literature rather than a lack of relevance.

Interestingly, no determinants were mapped to the policies and laws construct of the outer setting domain despite the fact that concerns about legal accountability, autonomy, and data security and privacy are often cited when discussing AI-based health care applications [8,27,31,35,36].

### Implications for Implementation Practice

The determinants identified in this review can be used to guide future implementation projects through the designation of appropriate strategies that mitigate barriers and leverage facilitators to successfully implement AI-based CDSSs. Our results support the use of several strategies derived from the Expert Recommendations for Implementing Change project, which aimed to synthesize knowledge from a wide range of implementation science and clinical practice experts [37].

First, developing and distributing educational materials explaining how the specific AI-based CDSS works, detailing the results of clinical trials or other external validation tests, and describing the patient population the system was developed with may help reduce barriers related to the innovation’s complexity and evidence base. Adopting an integrated knowledge translation approach that directly involves end users in both the design and implementation of new innovations could help mitigate these barriers further upstream [38]. Conducting educational meetings targeted toward different stakeholder groups, including clinicians, patients, caregivers, hospital administrators, and IT staff, to teach them about the innovation and its clinical benefit may also help reduce initial resistance to uptake [37].

End user resistance can also be eased through the identification of clinical champions; these individuals encourage uptake by raising awareness and providing motivation and leadership to their peers [37,39]. Uptake can be further encouraged through the influence of local opinion leaders, who should be identified and informed about the benefits of the innovation prior to its implementation [37].

Finally, implementation teams should aim to capture and share knowledge throughout the implementation process. Regular engagement sessions with clinician end users allowing for reflection on implementation efforts can be used to collect information about what specific strategies worked in their local context, which can later be disseminated to help inform future implementations of AI-based CDSSs [37].

### Comparison With Prior Work

The determinants identified in this scoping review, particularly those related to algorithm interpretability, data availability and quality, and workflow integration, are already well documented in the literature [40,41]. However, many of these studies identified barriers and facilitators based on anticipated implementation challenges prior to real-world deployment. Thus, our focus on accounts of real-world implementation represents a major strength of this scoping review, bridging the gap between theory and practice to provide actionable insights.

Furthermore, the determinants identified in this review are not limited to a single clinical domain, as is often the case with previously published work in this area [41,42]. Synthesizing determinants across a broader clinical context allows for more generalizable considerations that can be applied to a wider range of future CDSS implementations.

Recent interview-based work exploring stakeholder perspectives on improving AI-based CDSSs and their integration into care identified similar challenges related to workflow integration, evidence quality and transparency, clinician trust, training and education, and organizational readiness [43].

Although our findings are well supported, there are some determinants commonly cited in the literature that were not reported in the articles included in this scoping review. Notably, there was no mention of the effects of cost and willingness to pay, which are known to be key drivers of success for health care innovations [44]. This could potentially be a result of our focus on real-world implementations as these projects would likely have undergone a cost-benefit analysis prior to initiation at a given hospital or health system. However, it is important to note that AI-based CDSSs in particular require additional investments to deploy and maintain extensive IT infrastructure, as well as providing training for end users; these costs must be balanced by an improvement in the quality of patient care for implementation to be supported by hospitals and health systems [27,45,46].

Finally, consistent with our finding that only a limited number of studies explicitly reported implementation determinants, a recent scoping review examining patient-related benefits of AI-based CDSSs in sepsis care also emphasized the need for further implementation-oriented and prospective research to support real-world clinical integration [47].

### Limitations

This study has several limitations. First, this review adopted a broad definition of AI, encompassing both rule-based and data-driven systems. While this approach enabled the inclusion of a larger body of literature and a broader range of implementation determinants, the findings may not be specific to contemporary AI-based implementations. Therefore, some identified determinants may reflect broader CDSSs or health IT implementation considerations.

Second, our strict definition of clinical decision support and focus on real-world implementation studies resulted in a narrow scope and a limited number of included studies, which constrained the depth of analysis. As a result, unsuccessful, aborted, or prerollout implementation attempts may have been underrepresented despite their potential to provide important insights into barriers preventing real-world adoption. However, we feel that this approach was justified by our aim to address the knowledge gap pertaining to the real-world implementation of AI-based CDSSs. In addition, among the included studies, most did not report implementation outcomes in sufficient detail to identify determinants that were most influential to the successful adoption, integration, and sustained use of AI-based CDSSs over time.

Third, most included studies were conducted in the United States and within selected clinical contexts, which may limit the transferability of findings to other health care systems, organizational structures, and clinical settings.

Fourth, although our search strategy focused on major biomedical and health sciences databases, it is possible that relevant studies published in technical or engineering venues were not captured. Given our focus on real-world clinical implementation, we anticipate that most eligible studies would be indexed in clinically oriented databases.

Fifth, there was a limited representation of patient perspectives in the included studies. While this may partially reflect our focus on clinician-facing tools, understanding how patients perceive the use of AI-based CDSSs in their care remains an important area for future research.

Finally, this review is subject to temporal limitations. The literature search was conducted in 2022, and given the rapid evolution of AI in health care, additional relevant studies may have been published since that time. As such, the findings should be interpreted as reflecting the evidence base available at the time of the search.

### Conclusions

This review identified key determinants influencing the real-world implementation of AI-based CDSSs, emphasizing the importance of system design, organizational context, and implementation strategies. However, the limited number of studies reporting explicit implementation determinants underscores a critical gap in the literature. Future work should prioritize the systematic identification and reporting of implementation determinants to better inform adoption and sustainability. These findings provide a foundation for developing targeted implementation strategies and highlight the need for more rigorous, implementation-focused research to support the successful integration of AI-based CDSSs into routine clinical practice.

## Supplementary material

10.2196/89834 Multimedia Appendix 1 Supplementary tables showing an example full electronic search strategy and excerpts from the included studies.

10.2196/89834 Checklist 1 PRISMA-ScR checklist.

## Abbreviations

CDSS: clinical decision support system
CFIR: Consolidated Framework for Implementation Research
ML: machine learning
PRISMA: Preferred Reporting Items for Systematic Reviews and Meta-Analyses
PRISMA-ScR: Preferred Reporting Items for Systematic Reviews and Meta-Analyses extension for Scoping Reviews
