# Supplementary material for: Real-World Barriers to and Facilitators of Implementing AI-Based Clinical Decision Support Systems: Scoping Review
Source: JMIR Med Inform. 2026 Aug 13;14:e89834. doi: 10.2196/89834 (PMC13472529; doi:10.2196/89834)
Supplement: Supplementary file 2 [file medinform-v14-e89834-s001.docx]

**Supplementary Table S1**. Excerpts from studies describing determinants (barrier and facilitators) of implementing artificial intelligence–based clinical decision support systems in healthcare, mapped to CFIR domains and constructs.

| **Last Name of First Author** | **Excerpt** | **CFIR Domain** | **CFIR Construct(s)** | **Determinant** |
| --- | --- | --- | --- | --- |
| **Goldstein [13]** |  |  |  |  |
|  | “Academic informaticians, health services researchers, physician domain experts, and hospital information system specialists typically have disparate disciplinary perspectives and cultures. Consequently, uniting them on the same team with common overall objectives requires effort and commitment to develop a shared vocabulary and patterns for handing-off tasks. We approached this challenge by starting with small tasks and using bridge personnel, i.e., individuals who had familiarity with at least two of the four disciplines. As our team gained experience in working together, we developed patterns of communication and identified the most effective role assignments.” | Implementation Process | Teaming | Facilitator |
|  | “Early in the planning stage, we met with IRMS administrators and networking staff to outline our plans and obtain their input and approval, and we maintained close contact with the IRMS staff during planning and implementation. The IRMS staff provided support in several key areas including installing the project server in the IRMS server room so that it could benefit from backup power supply, air conditioning, and optimal network connections; programming the M patient data extract; and network support.” | Implementation Process | Planning | Facilitator |
|  | “Successful guideline implementation requires local clinical opinion leader ‘‘buy in’’ of the clinical content. Clinicians must be assured that the guideline recommendations are well founded. The recommendations presented should be based on sufficient backing.” | Innovation | Innovation Evidence Base | Barrier |
|  | “Both San Francisco and Durham had on-site physician-investigators who were primarily responsible for overseeing implementation, but neither of these physicians had special training or experience in informatics.” | Individuals | Implementation Leads, Capability | Barrier |
|  | “Since the VAPAHCS study site included clinics located in seven different cities across a wide geographic area, it was not feasible to bring the clinicians together for training." | Inner Setting | Access to Knowledge and Information | Barrier |
|  | “Clinicians receive many clinical reminders. One effective means to sustain clinician interest was to provide quarterly feedback on guideline–drug concordance for hypertension.” | Outer Setting | External Pressure (Performance Measurement Pressure) | Facilitator |
|  | “The physician-investigators in San Francisco and Durham were not the original developers of ATHENA DSS and did not initially have a sense of ‘‘ownership’’ of the knowledge base. Lack of familiarity with the program could potentially interfere with their enthusiastic endorsement of it to their colleagues.” | Individuals | Opinion Leaders, Motivation | Barrier |
|  | “Clinical opinion leaders must also be confident that the guidelines apply well to their own patient population. We recruited several physicians to assist with review of our guideline implementation in ATHENA DSS. In addition to the physician-administrators described above (one of whom was also the medical center’s overall guideline implementation leader), we recruited the supervisor of the general medical clinics at the Palo Alto site and the primary care chief resident as physician-monitors. We shared the knowledge rules used in ATHENA DSS, gave them individual training sessions in use of the system, activated the system at their clinics, and encouraged them to comment directly and to use the feedback features built into ATHENA DSS as described above.” | Implementation Process | Engaging | Facilitator |
| **Henry [14]** |  |  |  |  |
|  | "Clinicians described that their overall willingness to trust the ML-based system was rooted in several factors that helped them build a mental model of how the system worked. None of the clinicians fully understood the machine learning behind the system; however, while some were curious to learn more, they did not perceive that understanding the system's logic in an individual case would change their decision making [...] although unconcerned with the specific statistical model behind the system, clinicians reported having come to better understand how the system operated by observing its behavior in different scenarios and with different patient types." | Innovation | Innovation Complexity | Facilitator |
|  | "Clinicians valued external studies of the system and recommendations by colleagues and experts that allowed them to develop trust peripherally. [...] For instance, one ED physician said, 'I'd want to understand the population it was derived from...and then I'd want to see the population they validated it on afterwards...whether that group looks like the patients I'm treating.'" | Innovation | Innovation Evidence Base, Innovation Adaptability | Facilitator |
|  | "Clinicians also valued that they were able to ask questions about the system design choices during educational sessions and customize the interface and alert sensitivity to their environment and patient population [...] interacting with the deployment team also allowed for input into the tool's operations, which was described as an improvement over prior CDSS deployments." | Implementation Process | Assessing Needs (Innovation Deliverers) | Facilitator |
|  | "Concerns that regulatory agencies might use these systems to standardize care even in scenarios where a clinician disagrees with the system, potentially leading to over-treatment and patient harm, especially in cases where the alerts occurred prior to clinical recognition. [...] when asked what would convince them to act on the system's recommendations prior to apparent symptoms, suggestions included clinical trial evidence, or personally experiencing scenarios where the alert was dismissed but the patient was later diagnosed as having sepsis." | Outer Setting | External Pressure (Performance Measurement Pressure) | Barrier |
|  | "Ultimately, the capacity of state-of-the-art ML systems to improve clinical care depends on clinicians' ability and willingness to incorporate the information provided by these systems into their work." | Individuals | Innovation Deliverers, Motivation | Barrier |
| **Jauk [16]** |  |  |  |  |
|  | “The expert group appreciated that there was no need of additional data entry and that the prediction was available within few seconds in the user interface of the HIS.” | Inner Setting | Structural Characteristics (Information Technology Infrastructure) | Facilitator |
| **Ji [17]** |  |  |  |  |
|  | “...the AI + CDSSs in Chinese hospitals, similar to systems explored in other studies, may suffer from a mismatch between clinical needs and system functions thus making the absence of translating users’ requirements into system function design and clinical pathway integration one of the major obstacles faced by AI + CDSSs implementation. Therefore, it is recommended to [...] generate the right recommendations to the right person in the right intervention format through the right channel at the right time and location.” | Inner Setting | Compatibility | Barrier |
|  | “The second challenge related to the implementation of AI + CDSSs highlighted by our analysis is posed by data quality and availability. This is a common global problem where the lack of high quality and available data creates a barrier for practitioners to receive the “right information” from an AI + CDSS. The complex nature of medical data makes data quality problems more evident because missing, incorrect, or vague information may lead to useless or even harmful results.” | Inner Setting | Structural Characteristics (Information Technology Infrastructure) | Barrier |
|  | “The degree to which clinicians can understand how the AI + CDSS works and generates the information given and the basis for individual recommendations affects their trust in the system. The lack of methodological transparency inherent in many machine learning approaches (“black-box”) can impair user trust in the outputs produced by an AI + CDSS. This creates a distinctive phenomenon in the health care field where medical practitioners are less likely to adopt AI in clinical practice if they do not trust the technology or understand how it was used to support process of care or patient outcomes.” | Innovation | Innovation Complexity | Barrier |
| **Joshi [18]** |  |  |  |  |
|  | "Optimizing the alert to appropriately identify patients and trigger clinician response was the first major barrier to generating clinician acceptance. Optimizing the alert consisted of fine-tuning thresholds, content, and integration of the alert into the workflow. Themes focused on lack of consensus, tension between alert placement and disruption of workflow, and burden of optimization falling to individual institutions." | Inner Setting | Compatibility | Barrier |
|  | "The most frequently cited concern for clinician buy in for both rule-based and machine learning models was avoiding alert fatigue, or over-alerting users. A few remarked that this concern was present with all their decision support tools." | Inner Setting | Culture, Compatibility | Barrier |
|  | "Other themes focused on concerns about clinical relevance, difficulty explaining why models fire, confusion with understanding what an alert means, and distrust stemming from mismatched expectations." | Innovation | Innovation Evidence Base, Innovation Complexity | Barrier |
| **Miller [19]** |  |  |  |  |
|  | "Many providers suggested that education and consideration of workflow before and during implementation could facilitate programmatic success over time." | Implementation Process | Assessing Needs (Innovation Deliverers) | Facilitator |
| **Romero-Brufau [20]** |  |  |  |  |
|  | “One key facilitator of our ability to reduce hospital readmissions was our ability to identify patients at highest risk for hospital readmission. The observation that the risk reduction was highest in the high-risk subgroup suggests that interventions targeted toward high-risk patients ac- count for most of the change observed.” | Innovation | Innovation Relative Advantage | Facilitator |
| **Romero-Brufau [21]** |  |  |  |  |
|  | “The most commonly-listed facilitator was that the CDS promoted team dialog about patient needs (N = 14, 52 %). Five users (19 %) listed integration of recommendations into workflow and 3 users (11 %) listed usefulness of patient risk factors.” | Inner Setting | Compatibility | Facilitator |
|  | “The most commonly listed barriers were related to inadequacy of the interventions recommended by the CDS. Eleven users (24 %) were dissatisfied that the interventions were too similar between different patients and therefore were not sufficiently tailored to each patient. Eleven other users (24 %) felt that the suggested interventions were inappropriate or not useful. Eight users (18 %) were dissatisfied that the system inaccurately classified standard risk patients as high-risk (i.e., high false-positive rate)." | Innovation | Innovation Relative Advantage | Barrier |
| **Gonçalves [22]** |  |  |  |  |
|  | "The results of this experience are in the improvement of the performance of healthcare teams by the use of the tool, and the improvement of the tool itself. These potential benefits occur despite technological challenges, such as infrastructure in relation to computer terminals, bedside data recording equipment, tablets, smartphones and Wi-Fi networks; challenges of individual proficiency of health professionals in the use of hardware and software; and distrust in the accuracy of technology, among other challenges that influence the success of projects like this one." | Inner Setting | Structural Characteristics, Available Resources (Materials and Equipment) | Barrier |
|  |  | Individuals | Innovation Deliverers, Capability | Barrier |
|  |  | Innovation | Innovation Evidence Base | Barrier |
|  | "In the actual implementation process, the nurses act conduct an initial moment of awareness-raising with the healthcare teams, when they explain the process of conception of the tool, its mission, its functionalities, its impact on the nurses’ work process and the actions that are expected from the team so that the early identification of possible cases of sepsis is effective. Communicating the overall benefits of the implementation and of a new technology is fundamental to help the members of the staff – those who will effectively use the technology – to stay engaged and work together to achieve the success of a project. Among the skills to be built up by nurses in this implementation phase are communication, empathy, attentive listening, and technical knowledge about the tool and the nursing work process in that specific hospital context." | Implementation Process | Engaging | Facilitator |

**Supplementary Table S2.** Example full electronic search strategy

Database: MEDLINE (Ovid)
Search date: May 10^th^, 2022

| **Search Step** | **Search Query** |
| --- | --- |
| 1 | exp Artificial Intelligence/ |
| 2 | exp Machine Learning/ |
| 3 | exp Deep Learning/ |
| 4 | artificial intelligence.ti,ab,kf,tw. |
| 5 | machine learning.ti,ab,kf,tw. |
| 6 | deep learning.ti,ab,kf,tw. |
| 7 | augmented intelligence.ti,ab,kf,tw. |
| 8 | AI.ti,kf. |
| 9 | ML.ti,kf. |
| 10 | reinforcement learning.mp. |
| 11 | exp Neural Networks, Computer/ or neural network.mp. |
| 12 | exp Unsupervised Machine Learning/ or unsupervised learning.mp. |
| 13 | supervised learning.mp. or exp Supervised Machine Learning/ |
| 14 | random forest.mp. |
| 15 | exp Support Vector Machine/ |
| 16 | exp Decision Trees/ |
| 17 | exp Classification/ |
| 18 | rule-based.mp. |
| 19 | symbolic artificial intelligence.mp. |
| 20 | symbolic AI.mp. |
| 21 | 1 or 2 or 3 or 4 or 5 or 6 or 7 or 8 or 9 or 10 or 11 or 12 or 13 or 14 or 15 or 16 or 17 or 18 or 19 or 20 |
| 22 | exp Decision Support Systems, Clinical/ |
| 23 | decision support*.mp. |
| 24 | clinical decision support system.mp. |
| 25 | clinical decision support.mp. |
| 26 | evidence-based.mp. |
| 27 | evidence-based support.mp. |
| 28 | support system.mp. |
| 29 | exp Clinical Protocols/ |
| 30 | clinical guideline*.mp. |
| 31 | medical guideline*.mp. |
| 32 | medical guidance*.mp. |
| 33 | 22 or 23 or 24 or 25 or 26 or 27 or 28 or 29 or 30 or 31 or 32 |
| 34 | implement*.mp. |
| 35 | implementation strategy.mp. |
| 36 | strateg*.mp. |
| 37 | barrier*.mp. |
| 38 | facilitator*.mp. |
| 39 | enabler*.mp. |
| 40 | determinant*.mp. |
| 41 | satisfaction*.mp. |
| 42 | perception*.mp. |
| 43 | experience*.mp. |
| 44 | 34 or 35 or 36 or 37 or 38 or 39 or 40 or 41 or 42 or 43 |
| 45 | 21 and 33 and 44 |

*Note: This search strategy was adapted for other databases using database-specific subject headings and syntax.*
